# The distinct expression patterns of claudin-2, -6, and −11 between human gastric neoplasms and adjacent non-neoplastic tissues

**DOI:** 10.1186/1746-1596-8-133

**Published:** 2013-08-06

**Authors:** Zhe Lin, Xiaowei Zhang, Zhijing Liu, Qihui Liu, Liping Wang, Yan Lu, Yuanyuan Liu, Min Wang, Minlan Yang, Xiangshu Jin, Chengshi Quan

**Affiliations:** 1 The Key Laboratory of Pathobiology, Ministry of Education, Bethune Medical College, Jilin University, Changchun, Jilin, China; 2 Department of General Surgery, The Second Hospital of Jilin University, Changchun, Jilin, China

**Keywords:** Gastric cancer, Tight junctions, Claudin-2, Claudin-6, Claudin-11, Immunohistochemistry

## Abstract

**Background:**

Cancers have a multifactorial etiology a part of which is genetic. Recent data indicate that expression of the tight junction claudin proteins is involved in the etiology and progression of cancer.

**Methods:**

To explore the correlations of the tight junction proteins claudin-2,-6, and −11 in the pathogenesis and clinical behavior of gastric cancer, 40 gastric cancer tissues and 28 samples of non-neoplastic tissues adjacent to the tumors were examined for expression of claudin-2,-6, and −11 by streptavidin-perosidase immunohistochemical staining method.

**Results:**

The positive expression rates of claudin-2 in gastric cancer tissues and adjacent tissues were 25% and 68% respectively (*P* < 0.001). The positive expression rates of claudin-6 in gastric cancer tissues and adjacent tissues were 55% and 79% respectively (*P* = 0.045 < 0.05). In contrast, the positive expression rates of claudin-11 in gastric cancer tissues and gastric cancer adjacent tissues were 80% and 46% (*P* = 0.004 < 0.01). Thus in our study, the expression of claudin-2, and claudin-6 was down regulated in gastric cancer tissue while the expression of claudin-11 was up regulated. Correlations between claudin expression and clinical behavior were not observed.

**Conclusion:**

Our study provides the first evidence that claudin-2,-6, and −11 protein expression varies between human gastric cancers and adjacent non-neoplastic tissues.

**Virtual slides:**

The virtual slide(s) for this article can be found here: http://www.diagnosticpathology.diagnomx.eu/vs/5470513569630744

## Introduction

The progression of cancer is accompanied by multiple genetic and epigenetic alterations that have potential as markers for early diagnosis, treatment, and prevention [1]. Gastric cancer has become the second leading cause of cancer mortality worldwide accounting for almost 10% of newly diagnosed cancers [2]. Generally, early diagnosis of gastric cancer is difficult because patients tend to be asymptomatic until the tumors have reached an aggressive stage [3]. Accordingly, identification of specific early genetic markers of gastric tumorigenesis becomes significant. It has been reported that the endothelial lipase protein is a promising urinary biomarker for diagnosis of gastric cancer and is potentially applicable to general screening for cancer with high sensitivity and specificity [4]. Decreased Plakophilins (PKP2 and PKP3) may be early prognostic markers and loss of PKP3 expression during gastric carcinoma progression may indicate an invasive phenotype [5]. Moreover, the up regulation of differentiated embryonic chondrocyte-expressed gene 1 may play an important role in hypoxia regulation and cell proliferation in gastric cancer [6]. The detection of Ezrin expression can be used as a marker for early diagnosis and prognosis of gastric adenocarcinoma [7].

Tight junctions (TJs) are components of epithelial and endothelial cells that participate in the formation of intercellular junctional complexes. Tight junctions contribute to epithelial cell permeability, maintenance of cell polarity, and barrier function [8,9]. The claudin protein family is involved in formation of tight junctions (TJs), and consists of approximately 27 members, which are expressed with a tissue-specific distribution [10]. Malignant cells frequently display structural and functional disruption of the tight junctions [11]. Recently, the abnormal expression of members of the claudin protein family has been reported to participate in tumorigenesis [12]. For instance, claudin-3 and claudin-4 have been found to be regularly elevated in ovarian, breast, prostate and pancreatic tumors [13]. This observation suggests that alterations in claudin expression may occur as a common phenomenon related to human tumorigenesis and tumor progression. Moreover, claudin-4 has been reported to be highly unregulated in gastric cancer, with an association between the up-regulation of claudin-4 and lymph node metastasis [14]. Claudin-6 protein is significantly down-regulated in breast invasive ductal carcinomas and is an important correlate with lymphatic metastasis [15]. Together such observations suggest that claudin protein expression may be related to the survival and invasion of cancer cells and may have significant clinical relevance. However, to our knowledge, the exact expression patterns of the claudin protein family in gastric cancer have not been defined.

It has been reported that claudin-18 expression has been shown to have prognostic value in gastric cancer [16] and claudin-3,-4 and-7 expression are similarly elevated in gastric cancer [17]. Resnick et al. have determined that claudin-1,-3, and-4 and ZO-1 are strongly expressed in most gastric intestinal-type adenocarcinomas [18]. Strong expression of claudin-5 was associated with higher cell proliferation and apoptosis in gastric cancer [19]. In summary, in gastric cancer, claudin protein expression has been demonstrated to be of great importance and a relevant area for further study. Thus, the objective of this study was to examine the expression of claudin-2,-6, and −11 in gastric carcinoma and adjacent tissue which have been less well studied. We used immunohistochemical staining, and correlated the expression of these proteins with tumor differentiation and stage. One goal was to explore the feasibility of using claudin-2, -6, and −11 as potential prognostic markers.

## Materials and methods

### Patients

Paraffin blocks from forty specimens of gastric cancer and twenty-eight specimens ofhistologically normal tissue adjacent to the neoplasms were collected from patients being treated at the Second Hospital of Jilin University during the period between March 2011 and June 2011. The patients 'medical records were reviewed to determine their age and gender. Sections of the primary tumor were analyzed to identify the histological grade, and the presence or absence of regional lymph node metastasis. There were 31 men and 9 women with average age of 63 years. Eleven tumors had well differentiated histological appearance, another twenty-seven tumors were of moderately and poor differentiated. Whereas the remaining 2 cases were mucinous cyst-adenocarcinoma. For the use of these clinical materials for research purposes, prior patient’s consent and approval from the Institute Research Ethics Committee was obtained. All the cancer cases were classified and graded according to the International Union Against Cancer (UICC) staging system for gastric cancer.

### Materials

Rabbit antihuman claudin-2 antibody (BS1066), rabbit antihuman claudin-6 antibody (BS3107), rabbit antihuman claudin-11 antibody (BS1056) were purchased from Bioworld Technology (USA) and an streptavidin-perosidase immunohistochemistry reagent kit were purchased from Maixin Biology (Fujian, China).

### Immunohistochemistry

The sections were dewaxed by heating at 55°C for 30 min and subjected to two 15 min washes with xylene. Then, the sections were rehydrated by a series of 5 min washes in ethanol. The sections were placed into an enamel cylinder containing 10 mmol/L sodium citrate (pH 6.0), heated by gas cooker at 95°C for 5 min for antigen unmasking, and then were treated with 3% hydrogen peroxide for 30 min to inactivate endogenous peroxidase activity. After being incubated with fetal bovine serum for 30 min and sections were then incubated at 4°C overnight with rabbit anti-human claudin-2 antibody, rabbit antihuman claudin-6 antibody, or rabbit antihuman claudin-11 antibody diluted 1:400, 1:300, and 1:400 respectively. The sections were then washed with PBS and incubated for 30 min with biotinylated goat anti-rabbit secondary antibody at 37°C. The substrate, 3′3-diaminobenzidine tetrachloride, dissolved in steamed water, was added to visualize the positive expression. Negative control sections were immunostained as described above, but incubated with PBS instead of a primary antibody.

### Criteria for the positive claudin-2,-6 and-11 expression in tissue

The cells positively expressing claudin-2, -6, and-11 were identified by brown staining of their cytoplasm or cell membrane after reaction with claudin-2, -6, or −11 antibody. The claudin-2,-6,-11 positive tissues were quantified based on the percentage of positive cells which were serially counted in one microscopic field. The cell counting was repeated in five randomly-selected microscopic fields at × 400 magnification. The claudin-2 negative groups were defined as a field with level less than 20% (of the tumor cells); positive groups had more than 20% positive cells. The claudin-6 negative group had less than 15% stained cells and the positive group more than 15%. The claudin-11 negative group contained less than 30% positive cells and the positive group, more greater than 30%.

### Statistical analysis

The Chi-square test/Chi-Square Goodness-of-Fit Test was used to determine the prognostic significance value for disease progression of each factor alone, using a *P*-value < 0.05 for statistically significant associations. All the data were analyzed using SPSS 12.0 statistical software.

## Results

### Population and tumor characteristics

The clinicopathological characteristics of the patients are summarized in Table 1. Negative nodes were found in 15 cases; a total of 25 patients had positive metastatic nodes.

**Table 1:** **Expression of CLDN2**, **CLDN6**, **CLDN11 and clinicopathological characteristics in gastric cancer patients**

**Item**	**n**	**CLDN2(+)**	**CLDN2(-)**	***P***	**n**	**CLDN6(+)**	**CLDN6(-)**	***P***	**n**	**CLDN11(+)**	**CLDN11(-)**	***P***
Gastric cancer tissue	40	10	30	<0.001	40	22	18	0.045	40	32	8	0.004
Adjacent tissue	28	19	9		28	22	6		28	13	15	
Gender												
Male	31	9	22	0.404*	31	18	13	0.705*	31	26	5	0.645*
Female	9	1	8		9	4	5		9	7	2	
Age (year)												
≤60	17	4	13	1.000*	17	8	9	0.385	17	15	2	0.677*
>60	23	6	17		23	14	9		23	18	5	
Histological grade												
Well -differentiated	11	3	8	1.000*	11	6	5	1.000*	11	9	2	1.000*
Moderately and poor differentiated	27	7	20		27	15	12		27	21	6	
Lymph node metastasis												
+	25	7	18	0.715*	25	14	11	0.870	25	19	6	0.224*
-	15	3	12		15	8	7		15	14	1	

^*^ No statistical significance.

### The expression of claudin-2 and claudin-6 was reduced in gastric cancer

**Figure 1:**
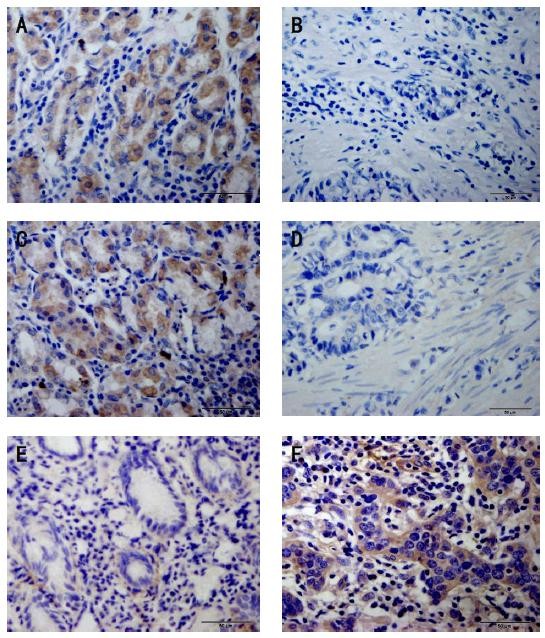
**Immunohistochemical demonstration of claudin protein expression in human gastric cancer and adjacent tissue.** Claudins were expressed in the cell cytoplasm and membrane. **(A)**, claudin-2 was highly expressed in epithelial cells adjacent to gastric cancer but was expressed at low levels in cancer tissue itself **(B)**. **(C)**, the strong expression of claudin-6 in tissue adjacent to human gastric cancer compared to low expression of claudin-6 in human gastric cancer tissue **(D)**. **(E)**, low claudin-11 expression was detected in tissue adjacent to human gastric cancer compared with high claudin-11 expression in human gastric cancer tissue **(F)** (400×).

In our study, claudin-2 expression was evaluated in the cytoplasm or membranes of 40 gastric cancers tissues and 28 specimens containing gastric tissue adjacent to the carcinoma. Positive expression of claudin-2 protein was found in 25.0% (10/40) of gastric carcinoma tissues and in 67.8% (19/28) of adjacent tissues (Table 1). The expression of claudin-2 in gastric cancer tissues was significantly lower than in adjacent tissues (The Chi-square test/Chi-Square Goodness-of-Fit Test, *P* < 0.001) (Figure 1A,B). As shown in Table 1 the expression of claudin-2 was not correlated with age (*P* =1.000), sex (*P* =0.404), histological grade (*P* = 1.000), or lymph node metastasis (*P* = 0.715).

Positive expression of claudin-6 protein was found in 55.0% (22/40) of gastric cancer tissues and in 78.6% (22/28) of adjacent tissues (Table 1). The expression rate of claudin-6 in gastric cancer tissues was lower than the rate in adjacent tissues (The Chi-square test/Chi-Square Goodness-of-Fit Test, *P* =0.045 < 0.05) (Figure 1C,D). As shown in Table 1, the expression of claudin-6 was also not correlated with age (*P* =0.385), sex (*P* =0. 705), histological grade (*P* = 1.000), or lymph node metastasis (*P* = 0.870).

### The expression of claudin-11 was increased in gastric cancer

The cytoplasmic staining of claudin-11 was strong in gastric cancer tissues and weak in adjacent tissues. Claudin-11 was expressed in 80.0% (32/40) of gastric cancer tissues. Cells were positive for claudin-11 in 46.4% (13/28) of tissues adjacent to the cancer. We conclude that claudin-11 expression is significantly higher (Figure 1E,F) in gastric cancer samples than in histologically normal gastric tissue. (The Chi-square test/Chi-Square Goodness-of-Fit Test, x2 = 8.293, *P* < 0.01). The expression of claudin-11 was not correlated with age (*P* =0.677), sex (*P* =0. 645), histological grade (*P* = 1.000), or lymph node metastasis (*P* = 0.224).

### Claudin-2 and claudin-6 may be concurrently expressed in gastric cancer

We investigated the correlation between claudin-2, claudin-6 and claudin-11 expression using The Chi-square test/Chi-Square Goodness-of-Fit Test. Although we did not find a correlation between claudin-11 and claudin-2 (The Chi-square test/Chi-Square Goodness-of-Fit Test, φ = 0.168, *P* = 0.405) or with claudin-6 (The Chi-square test/Chi-Square Goodness-of-Fit Test, φ = 0.176, *P* = 0.430), we found that the expression of claudin-2 was positively correlated with the expression of claudin-6 (The Chi-square test/Chi-Square Goodness-of-Fit Test, φ =0.376, *P* = 0.028). The detailed results of the analysis are described in Tables 2 and 3.

**Table 2:** **Correlation between the expression of claudin**-**6 and claudin**-**2**

**Item**	**CLDN2(+)**	**CLDN2(-)**	φ*	***P***	**CLDN11(+)**	**CLDN11(-)**	φ*	***P***
CLDN6(+)	8	11	0.376	0.028*	19	3	0.176	0.430
CLDN6(-)	2	19			13	5		

*φ Phi coefficient.

**Table 3:** **Correlation between the expression of claudin**-**2 and claudin**-**11**

**Item**	**CLDN11(+)**	**CLDN11(-)**	φ*	***P***
CLDN2(+)	10	1	0.168	0.405
CLDN2(-)	22	7		

*φ Phi coefficient.

## Discussion

Currently, the disruption of claudins expression is regarded as one of the mechanisms responsible for loss of cell adhesion, altered polarity, poor differentiation and increased invasive potential of neoplastic cells [20-23]. Although the normal ratio of claudins protein has a role in maintaining the structure and function of tight junctions in epithelial cells [24], the mechanisms by which claudin expression and destruction of tight junctions induce tumor formation and the effect of these changes on tumor progression have not been studied in detail. It has been postulated that both abnormal up-regulation and down-regulation of claudin proteins would cause the structural and functional disruption of tight junctions, for instance, destruction of tight junction integrity, alteration of intercellular space, and weakening of tight junction cohesion [25]. In addition, the alteration of claudins protein expression can regulate cellular proliferation, differentiation, survival, and apoptosis through a series of signal transduction pathways, thus, playing an important role in tumorigenesis and tumor metastasis [26,27]. Claudin-4 has been shown to activate MMP-2 and claudin-4 expression has been significantly associated with MMP-9 expression, indicating that claudin-mediated increased cancer cell invasion result from activation of MMP proteins [28]. Phosphorylation of claudin-3 by cAMP-dependent protein kinase and claudin-4 by Ephrin Type-A Receptor 2 can modulate cell-to-cell contact [29,30]. Claudin-1 is involved in the β-catenin- T-cell Factor/ Lymphoid Enhancing Factor signaling pathway, and increased expression of claudin-1 may be a component of colorectal tumorigenesis [31]. It has been reported that Claudin-7 unlike other claudins, has both structural and regulatory functions and may be related to cell differentiation [32]. Alteration of claudin expression may affect permeability at tight junction, possibly increasing the diffusion of nutrients and other extracellular growth factors to promote cancer cell growth, survival and motility in gastric cancer [33]. In brief, claudin proteins may participate in regulation of cell proliferation, differentiation and apoptosis directly and indirectly [34].

Recently, claudin-2 has been reported selective up-regulated in colorectal cancer and may be useful as tumor markers and targets for the treatment of colorectal cancer [35]. Nevertheless, claudin-2 protein expression was significantly down-regulated in tumors compared with corresponding normal breast tissue. Down-regulation of claudin-2 was significantly associated with lymph node metastasis in breast carcinomas by Western blot analysis, and with high clinical stage by immunohistochemistry [36]. Similarly, claudin-2 were selective down-regulated in gastric cancer compared with corresponding cancer adjacent tissues in our present data. However, the association between claudin-2 protein expression with high clinical stage and lymph node metastasis has not been observed.

We cloned putative mammary cancer suppressor (*mes*) gene claudin-6 in mammary epithelial cells purified from Cop rat that extremely resistant to mammary cancer reduced by a variety of carcinogen. We have also reported that up-regulation of claudin-6 may induce apoptosis and decrease clone formation, invasiveness and migration of MCF-7 in vitro [37]. Epigenetic silencing of claudin-6 promoted anchorage-independent growth, cellular invasiveness and transendothelial migration of breast carcinoma cells, accompanied by an increase in matrix metalloproteinase activity [38]. It is reported that apoptosis signal-regulating kinase 1 is associated with the effect of claudin-6 in breast cancer [39]. Recent gene expression microarray analyses have indicated that claudin-6 is specifically expressed in atypical teratoid rhomboid tumors (AT/RTs), suggesting a role as a positive diagnostic marker of AT/RTs [40]. On the contrary, in the present study we found that claudin-6 protein wan expressed at low levels in gastric carcinoma tissues but highly expressed in histologically normal adjacent tissues.

Claudin-11, an oligodendrocyte protein, has been shown to interact with α1-integrin and to regulate the proliferation and migration of oligodendrocytes in culture [41]. Loss of claudin-11 may be considered to be putative indicators of recurrence and more aggressive behavior of meningiomas [42]. Accordingly, the overexpression of claudin-11 would decreases the invasive potential of bladder cancer cells in vitro [43]. However, in our present work the cytoplasmic staining of claudin-11 was strong in gastric cancer tissues and weak in adjacent tissues, reveals that claudin-11 may be a positive diagnostic marker in gastric cancer which was different with claudin-2 and claudin-6. In addition, recent data reveals that claudin-16 and claudin-19 interact and form a tight junction complex generated cation selectivity of the TJ in a synergistic manner [44].

Our present data observes that claudin-2 and claudin-6 were both down-regulated and may be concurrently expressed in gastric cancer, reveals that claudin-2 and claudin-6 may act as synergistic tumor suppressors in gastric cancer. Nevertheless, the correlations between claudin-11 expression with claudin-2 and claudin-6 expression have not been observed.

## Conclusion

The present work infers that the expression altered of claudin-2, claudin-6, and claudin-11 between human gastric cancers and adjacent non-neoplastic tissues and does not correlate with their clinical behavior. In addition, claudin-2 and claudin-6 may be concurrently expressed in gastric cancer. However, the specific mechanism responsible for these observations needs to be addressed in the future.

## Competing interests

The authors declare that they have no competing interests.

## Authors’ contributions

CQ carried out part of experiments, participated in the design of the study, performed the statistical analysis, and drafted the manuscript. XZ, ZL and ZL carried out most of experiments, and helped draft the manuscript. QL, LW, MW, YL, YL and YL assisted with the experiments, and helped to edit the paper. All authors have read and approved the final manuscript.
